# Self-Complexity and Self-Concept Differentiation – What Have We Been Measuring for the Past 30 Years?

**DOI:** 10.1007/s12144-014-9285-7

**Published:** 2014-11-09

**Authors:** Aleksandra Pilarska, Anna Suchańska

**Affiliations:** Department of Personality Psychology, Institute of Psychology, Adam Mickiewicz University, Szamarzewskiego 89, 60-568 Poznań, Poland

**Keywords:** Self-complexity, Self-concept differentiation, Measurement, Identity, Cognitive processing

## Abstract

Research on the relation between the structure of the self-concept and psychological well-being has yielded seemingly inconsistent and even conflicting results. This article presents studies that examined the validity of often-used measures of self-complexity and self-concept differentiation and tested their ability to predict personal identity and active cognitive processing. The findings revealed several conceptual and methodological problems that continue to plague self-structure research, including the conflating of self-concept content and self-concept structure. In short, our data indicated that the commonly used indices of self-complexity and self-concept differentiation cannot be considered pure measures of the underlying dimensions of self-structure. In addition, only weak correlations of the self-structure variables with measures of personal identity and thinking dispositions have been found. Moreover, once the theoretically irrelevant sources of variance were controlled, the effects of the included structural features of the self-concept on the outcomes of interest either did not occur or were less pronounced. Given the above, it seems reasonable to suggest that at least some of the conclusions regarding the adaptive value of self-structural variables drawn from previous research in this field need revision.

## Introduction

The majority of contemporary theorists and researchers in personality psychology agree that self-concept is a dynamic and multifaceted phenomenon (e.g., Greenwald and Pratkanis 1988; Markus and Wurf 1987; Roberts 2007; Suszek 2007; Swann and Bosson 2010). The notion of the self as plural allows distinguishing between its content (i.e., what one thinks one is like) and structural features (i.e., how the contents are organized). After nearly 3 decades of research it has been shown that there are individual differences in the self-concept structure. However, the functionality of various structural features of the self-concept appeared to be different depending on their operationalization and measurement.

This article presents the results of a series of studies carried out by the authors, sharing a common focus on the validity of operationalization and adaptive value of two, broadly discussed in the psychological literature, structural aspects of self, namely self-complexity (Linville 1985, 1987) and self-concept differentiation (Donahue et al. 1993).

### Self-Complexity

Among the various models of self-complexity (e.g., Anderson 1992; Evans 1994; Stein 1994; Woolfolk et al. 1995), Linville’s social-cognitive model of self-complexity (1985, 1987) is by far the most widely studied and cited (Rafaeli and Hiller 2010). According to Linville, self-complexity reflects the number and diversity of self-aspects developed for meaningful aspects of one’s life. So, theoretically, it is considered as two-dimensional, capturing both differentiation and integration. To measure self-complexity, Linville adopted the dimensionality statistic (H), a measure of nominal scale dispersion utilized in exact sciences. This index is obtained in a trait-sorting task in which participants ascribe a predefined set of traits to a variable (self-generated) set of self-aspects. High self-complexity results from trait sorts composed of a high number of self-aspects with low redundancy between them (i.e., low repetition of trait adjectives). The basic assumption of Linville’s model (1985, 1987) is that greater self-complexity moderates the negative effects of stressful situations. With a more complex self-concept, the impact of a stress-inducing event is less likely to spill over from one self-aspect to another.

Research examining the stress-buffering role of self-complexity has been vigorously pursued since Linville’s model was introduced. Over the decades, it has been used to address various adjustment outcomes, ranging from depression (e.g., Brown and Rafaeli 2007; Linville 1987) to narcissism (Rhodewalt and Morf 1995). These studies, however, have yielded mixed results. Specifically, some research has shown a positive link between self-complexity and psychological adjustment (e.g., Cohen et al. 1997; Dixon and Baumeister 1991; Linville 1987; Niedenthal et al. 1992), some studies have found a negative link (e.g., Brown and Rafaeli 2007; Jordan and Cole 1996), and some findings implied no relationship (e.g., Assanand 2003; Campbell et al. 2003; Gramzow et al. 2000). Such results have called into question both the validity of Linville’s measure and the model itself (see, for review, Koch and Shepperd 2004; Rafaeli and Hiller 2010; Rafaeli-Mor and Steinberg 2002). Several researchers argue that different types of content could be organized differently and that such factors as the valence of self-descriptors should be taken into account (Morgan and Janoff-Bulman 1994; Woolfolk et al. 1995). Other studies suggest, though, that what is problematic is not the model, but its measurement, namely the H statistic (Locke 2003; Rafaeli-Mor et al. 1999). As a result, distinguishing two components of self-complexity – the number of self-aspects and the degree of overlap in their content – has been advocated as a more appropriate approach (Rafaeli-Mor et al. 1999). Based on these two measures, a new composite measure of self-complexity has then been proposed (SC, Sakaki 2004).

### Self-Concept Differentiation

The second component of self-complexity, overlap, can be conceptually tied to the concept of self-concept differentiation (self-incoherence).^1^ Self-concept differentiation refers to an individual’s tendency to view oneself as possessing different personality characteristics across different social roles or contexts (Donahue et al. 1993). To assess self-concept differentiation, Donahue et al. (1993) developed the procedure in which participants rate how well a fixed set of traits describe them in experimenter-provided social roles. The self-concept differentiation index (SCD) can be expressed as the unshared variance, the mean intercorrelation, or the absolute differences among the role identities (Campbell et al. 2003; Donahue et al. 1993; Styła et al. 2010). Various linear transformations of the above indices are also used, although less frequently (e.g., Sheldon et al. 1997; Suh 2002).

Prior research on self-concept differentiation has pointed to the adaptive value of self-concept consistency and argued that high self-concept differentiation is indicative of an incoherent and fragmented self-concept (e.g., Campbell et al. 2003; Diehl and Hay 2010; Diehl et al. 2001; Donahue et al. 1993; McReynolds et al. 2000; Sheldon et al. 1997; Styła et al. 2010). This concurs with Block’s (1961) description of an individual who lacks a coherent self as “an interpersonal chameleon, with no inner core of identity, fitfully reacting in all ways to all people” (p. 392). Although the association of self-concept differentiation with psychological maladjustment has been repeatedly demonstrated, it might appear contrary to both everyday and clinical experience. After all, rigidity and inflexibility of behavior is considered as an essential diagnostic criterion for personality disorders (DSM-IV, American Psychiatric Association 1994; ICD-10, World Health Organization 1992). Additional concerns were raised by findings that the SCD index, while in theory a measure of the self-concept structure, was influenced by its content (Locke 2006). Moreover, the indices based on correlation coefficients do present some calculation problems, since they are also dependent upon variance in how traits are rated in a given situation (Baird et al. 2006; Locke 2006). In fact, the SCD index will not be identified if there is no within situation variance whatsoever. Finally, methodological concerns have been raised with regard to the SCD index based on an average standard deviation. Specifically, Baird et al. (2006) argued that this measure of self-concept differentiation conflates trait mean-level information and information concerning within-person variability, and that the previously identified associations between psychological adjustment and self-concept differentiation may be, at least to some degree, a statistical artifact (see also La Guardia and Ryan 2007).

### Self-Complexity Versus Self-Concept Differentiation

Since both self-complexity – overlap in particular – and self-concept differentiation theoretically refer to the same idea of contextualized self-views, how they relate to each other has received some attention. Donahue et al. (1993) argued that these aspects of self-concept structure are different in two respects: first, self-complexity includes not only the distinctiveness, but also the numerousness of self-aspects; and second, the original Linville’s self-complexity hypothesis does not postulate a direct link between self-complexity and adjustment. Campbell et al. (1991) contributed to this issue by pointing out that there is a crucial, though slightly ambiguous, difference between a complex self-concept and an uncertain one. In a similar way, Koch and Shepperd (2004) emphasized the distinction between self-complexity and cohesion within the self.

The empirical evidence supports the above reasoning by indicating that self-complexity and self-concept differentiation have dissimilar relationships with indices of well-being. Specifically, measures of self-concept differentiation have consistently been found to be positively related to depression, negative affectivity, and low self-esteem, whereas self-complexity measure has been shown to buffer against the harmful effects of stress on mental health outcomes such as depression (e.g., Campbell et al. 2003; Constantino et al. 2006; Dixon and Baumeister 1991; Lutz and Ross 2003; Niedenthal et al. 1992). Perhaps more puzzling is the lack of convergence of indices that purport to measure only the distinctiveness component of self-concept structure (also referred to as self-concept unity), namely overlap and self-concept differentiation (e.g., Constantino et al. 2006; Diehl et al. 2001). Such findings are, however, consistent with the suggestion that measures of SCD tap the negative experience of a fragmented self, whereas measures of self-complexity tap the positive experience of specialization of role identities. This difference supposedly is due to the way the two constructs are assessed – self-complexity scores are derived from self-generated roles that are idiosyncratic in content, whereas self-concept differentiation task uses participants’ self-ratings for personality attributes across experimenter-provided roles (e.g., Donahue et al. 1993; Koch and Shepperd 1991, Lutz and Ross 2003).

### Summary and Aim

There is a vast body of literature – theoretically, empirically, and clinically anchored – that suggests the importance of considering self-structure variables when attempting to understand the nature and function of the self-concept. Yet, empirical work in this area has produced inconsistent findings and is clearly hampered by measurement problems. This article aims to address these issues. Specifically, the purposes of the studies were to:

- examine different measures of self-complexity, obtained from the same trait-sort task: the quantity of self-aspects and the overlap among them, each reflecting one of the self-complexity components (Rafaeli-Mor et al. 1999), and the H statistic (Linville 1987) and the SC statistic (Sakaki 2004), each representing a singular measure of self-complexity;
- examine different indices of self-concept differentiation: one representing the absolute differences among the identities (i.e., the mean standard deviation across attributes; Donahue et al. 1993; Styła et al. 2010), one expressed as the mean intercorrelation among the role identities (Campbell et al. 2003), and one representing the proportion of variance in the role-identity ratings that was not shared across the roles (Block 1961; Donahue et al. 1993);
- investigate the mutual relationships between various measures of self-complexity and self-concept differentiation;
- test the assumption of independence of self-concept structure and self-concept content, specifically the sensitivity of self-complexity and self-concept differentiation indices to affective valence of self-descriptions used to measure them; and
- establish the relationship between self-complexity, self-concept differentiation, and well-being outcomes, particularly those relating to personal identity and thinking dispositions.

Our focus on personal identity stems from the notion, well grounded in theoretical and empirical research, that regards a sense of personal identity as an indication of effective adaptation and mental health, the cornerstone of the capacity to do well (e.g., Crawford et al. 2004; Erikson 1980; for review, see also Bosma and Kunnen 2001; Schwartz 2001). In addition to the identity variables, we also chose to focus on three cognitive variables that are known to promote meaning and integration of self-knowledge: need for cognition (Cacioppo et al. 1996), reflection (Trapnell and Campbell 1999), and integrative self-knowledge (Ghorbani et al. 2008). Each of these variables has displayed positive associations with psychological adjustment (e.g., Cacioppo et al. 1996; Fleischhauer et al. 2010; Ghorbani et al. 2010, 2008; Trapnell and Campbell 1999), contributing to a growing consensus that self-awareness and self-understanding play a key role in self-regulation. This examination becomes even more important, since structural features of the self-concept themselves reflect the ways that people cognitively organize knowledge about the self (e.g., Linville 1985; Rafaeli-Mor and Steinberg 2002).

It should be noted that, despite the fact that self-complexity and self-concept differentiation have been extensively researched, their relationships with personal identity and thinking dispositions have received little attention. To the extent that the existing literature allows conclusions, it appears that the greater the differentiation and the lower the integration among one’s self-aspects, the weaker one’s sense of identity (e.g., Block 1961; Donahue et al. 1993; Goldman 2004). However, that postulation still needs to be explored empirically. As for thinking dispositions in question, we are not aware of any empirical evidence regarding the relationships of these factors to self-complexity and self-concept differentiation.

## Method

### Overview and Procedure

The data reported in this paper were drawn from three studies carried out as part of a research project aimed at identifying relationships between self-concept structure and characteristics of personal identity. The studies were conducted in a collaborative mode, ensuring anonymity and confidentiality. The participants were informed about the purpose of the research project. The consent of each individual was the condition of participation in the research. Participants completed measures of self-complexity, self-differentiation, identity, and thinking dispositions as well as several questionnaires assessing constructs not relevant to the subject of this article.

### Participants

The samples were pooled based on age and education. We utilized data collected from university students and young adults with a college degree. In Study 1a there were 336 participants, whose ages ranged from 18 to 35 (*M* =21.65 yrs, *SD* =3.20 yrs). Women comprised 65.8 % of the participants. Study 1b included 544 participants between the ages of 18 and 32 (*M* =21.26 yrs, *SD* =1.46 yrs). Of this sample, 59.4 % were female. The sample in Study 1c consisted of 131 participants whose ages ranged from 20 to 34 (*M* =24.66 yrs, *SD* =3.13 yrs); 54.3 % were female.

### Measures

Because the three studies differed in the content of the test batteries administered, we note below which studies contained each measure.

#### Self-Complexity

To obtain measures of self-complexity, we used the Self-Complexity Questionnaire by Barczak et al. (2007). This instrument is based on self-descriptive trait-sorting task used by Linville (1987), but contains several minor adaptations.

First, the trait adjectives were listed in a sheet given to each participant, instead of being printed on cards. Second, a longer and more balanced list of traits was used. Sixty traits, divided equally between those with positive and negative valence, were used in place of the original list of 33 (mostly positively valenced) traits. The stimulus words were compiled by having psychology students (*N* = 186) provide adjectives that described eight people: a teacher they like, a politician they wouldn’t vote for, their best friend, a sibling, a person they don’t like, their ideal person and the opposite of that ideal. This resulted in a list of 493 unique adjectives. The final list was composed of 30 positively valenced adjectives (e.g., outgoing, trustworthy, kind, ambitious) and 30 negatively valenced adjectives (e.g., mean, selfish, lazy, withdrawn) most commonly mentioned by the students.

The participants were provided with a list of adjectives and a recording sheet with blank columns. They were first prompted to read the list and then think of the different roles they play in their lives. After that the participants were asked to form groups of traits, so that each group was descriptive of an aspect of their life. The descriptive groups were recorded in the blank columns of the recording sheet and labeled by the participants. No limit was placed on the number of groups or on the number of adjectives within each group. The participants were informed that each adjective may be used in more than one group or not at all.

Each participant’s trait sort was then used to calculate four measures of self-complexity. Two of them indicated the two components of self-complexity as suggested by Rafaeli-Mor et al. (1999). One was the number of self-aspects formed by the participants (NSA). The other was the degree of overlap between self-aspects (OL), which reflects the average communality between all pairs of self-aspects. Another two measures represented self-complexity as a whole. One was the H statistic endorsed by Linville (1987), which represents the number of independent or nonredundant dimensions underlying each grouping. The other was the SC statistic proposed by Sakaki (2004), which is a single composite measure of self-complexity, alternative to the one of Linville (1987). As argued by Sakaki (2006), the SC statistic provides a direct index of self-complexity, and serves as a better indicator of self-complexity than the H statistic. The corresponding formulas are presented in Table 1. A remark should be made regarding Sakaki’s SC formula, which uses overlap as a divisor. If the overlap score is zero then the result would be a divide by zero error. Unfortunately, Sakaki (2004) did not comment on this or propose an alternative approach to avoid the divide-by-zero situation.

**Table 1:** Formulas for calculating self-complexity

*OL* = (∑_i_(∑_j_ *C*_ij_)/T_j_)/*n* * (*n* − 1), where *C* is the number of common features in two aspects; *T* is the total number of features in the referent aspect; *n* is the total number of aspects in the person’s sort and *i* and *j* vary from 0 to *n* (*i* and *j* unequal).
*H* = log_2_ *n* − (∑_i_ *n*_i_ log_2_ *n*_i_)/*n*, where *n* is the total number of features (here 60), and *n*_i_ is the number of features that appear in a particular group combination.
*SC* = *NSA*/*OL*, where *NSA* is the total number of self-aspects in the person’s sort and *OL* is the person’s overlap score.

The Self-Complexity Questionnaire was included in test batteries administered in Studies 1b and 1c.

#### Self-Concept Differentiation

The participants completed the Self-Incoherence Scale by Styła et al. (2010). This tool is a self-concept integration measure based on Block’s (1961) and Donahue et al.’s (1993) scales. The participants were instructed to rate how descriptive 7 personality traits are of them in each of five different social roles (student, romantic partner, son or daughter, friend, and worker), using a 7-point Likert scale.

The 7 attributes (i.e., active, open-minded, loyal, self-confident, resourceful, independent, direct) were selected after a series of pilot studies. First, a group of psychology students (*N* = 24) was asked to read the list of 68 trait adjectives and mark those which describe them. An initial pool of attributes was derived from the Questionnaire of Social Perception (Jarymowicz 2008). They were broad personal characteristics and adjective markers that represent the Big Five traits (e.g., creative, outgoing, hardworking, helpful, sensitive). The pilot version of the Self-Incoherence Scale included 27 adjectives most commonly chosen by the students. In succeeding studies, a total of 317 participants completed the pilot version. Then, all items were factor-analyzed to obtain factor scores for each of them. Fourteen items with the greatest factor loadings were selected and split into two parallel versions of the scale. A final study (*N* = 94) showed that one of these versions produced better validity coefficients then the other, and was suggested as preferable for scientific research (Styła et al. 2010).

Three different indices of self-concept differentiation were computed for each participant from the data generated by this task. The first one was suggested by Styła et al. (2010), but previously used also by others (e.g., Donahue et al. 1993; Goldman 2004). This index represents the absolute differences among the roles. In particular, we computed the standard deviation of each of the participant’s personality trait ratings across each role (7 standard deviations in all), and then averaged them. The resulting score (SCD_SD_) represents the extent that participants’ personality trait ratings had deviated from one another when describing themselves across their different roles. As the second index of self-concept differentiation we used the average correlation among the roles (SCD_R_), as proposed by Campbell et al. (2003). Correlations between each participant’s five roles (10 correlations in all) were computed on the basis of the adjectives ratings made in each role. This measure provides an inverse measure of self-concept differentiation. The third index was computed by factor-analyzing the correlation matrix, and subtracting the percent of variance accounted for by the first principal component from 100 percent. The resulting score represents the proportion of unshared variance among the roles (SCD_VAR_). That is, higher scores on this measure reflect greater differentiation of self. This index was proposed by Block (1961), and then used by Donahue et al. (1993).^2^

The Self-Incoherence Scale was used in Studies 1a and 1b.

#### Sense of Identity

To measure a sense of personal identity, understood as a recurring mode of experiencing oneself-as-subject, extended form of the Multidimensional Questionnaire of Identity (MQI; Pilarska 2012, 2014a) was employed. The questionnaire consists of six subscales measuring the degree of accessibility, specificity, separateness, coherence, stability, and valuation of identity content (referred to, respectively, as sense of having inner contents, sense of uniqueness, sense of one’s own boundaries, sense of coherence, sense of continuity over time, and sense of self-worth), including a total of 43 items (e.g., I feel that I was once a very different person than I am now; It happens that I perceive my close one as an important part of my self). All items are evaluated on a four-level scale ranging from “strongly disagree/never” to “strongly agree/always”. In addition, a single composite score for a global sense of identity (GSI) was computed by averaging scores across all identity dimensions.^3^ In earlier studies, reliability coefficients for the identity dimensions varied from 0.62 to 0.86, with an average Cronbach’s alpha of 0.74 (e.g., Pilarska 2014a; Suchańska and Worach 2013). For our sample, the standardized Cronbach’s alpha coefficient for the overall scale was 0.90, and ranged from 0.62 to 0.79 (average, 0.72) for the individual subscales.

We included the Multidimensional Questionnaire of Identity in all three studies.

#### Identity Processes

The Identity and Experience Scale (IES) by Whitbourne and collegues (2002) in its Polish version by Suchańska and Jawłowska (2010, as cited in Jawłowska 2010) was used for measuring the identity processes. This tool consists of 33 statements, 11 for each scale: assimilation (e.g., When it comes to understanding myself, I’d rather not look too deeply), accommodation (e.g., Very influenced by what others think), and balance (e.g., Often take stock of what I have or have not accomplished). The participants respond on a seven-point scale from “definitely no” to “definitely yes”. The three subscales demonstrated reasonable internal consistency, with a standardized Cronbach’s alpha of 0.69 for assimilation, 0.83 for accommodation, and 0.83 for balance.

The Identity and Experience Scale was employed in Study 1c.

The Study lb battery contained the following scales measuring selected thinking dispositions.

#### Need for Cognition

Need for cognition was assessed via an adapted version of the Need for Cognition Scale (NCS; Cacioppo and Petty 1982; Matusz et al. 2011). The scale includes 36 items that focus on engagement in and enjoyment of intellectual activities (e.g., I try to avoid situations that require intensive thinking from me; I enjoy broadening my knowledge about things); each evaluated on a five-point scale, ranging from “strongly disagree” to “strongly agree”. Psychometric properties of the Polish version of the NCS are comparable to those of the original version: the reliability and stability indices are α = 0.91 and *r* = 0.86, respectively (Matusz et al. 2011). In the present sample, the internal consistency of this NCS, as measured by Cronbach’s standardized reliability coefficient, was α = 0.88.

#### Reflection

Reflection, an openness-related form of self-focused attention, was measured with the 8-item Reflection subscale taken from the Rumination-Reflection Questionnaire – Shortform (RRQ Shortforms) by Trapnell (1997). Every item (e.g., I love exploring my “inner” self) is presented on a five-point scale, allowing for a range of responses from “strongly disagree” to “strongly agree”. The Cronbach’s standardized reliability coefficient for the translated version of this scale was 0.80.

#### Integrative Self-Knowledge

The Integrative Self-Knowledge Scale (ISK; Ghorbani et al. 2008; Polish adaptation by Pilarska 2014b) assesses a temporally integrated understanding of processes within the self. The scale includes 12 items referring to an individual’s efforts (1) to understand past experience (e.g., If I need to, I can reflect about myself and clearly understand the feelings and attitudes behind my past behaviors), (2) to maintain awareness of the self in the present (e.g., Most of the time, I get so involved in what is going on that I really can’t see how I am responding to a situation), and (3) to move toward desired goals in the future (e.g., By thinking deeply about myself, I can discover what I really want in life and how I might get it). Each item is rated on a five-point Likert scale, ranging from “largely untrue” to “largely true”. The Polish version of the ISK scale has good construct validity, and satisfactory internal consistency (Pilarska 2014b). In our sample, internal reliability in terms of Cronbach’s standardized alpha was found to be 0.79.

## Results

For clarity of presentation, the results with brief comments are presented in five major sections, followed by a more general discussion of the results and their implications. It should be noted that parts of the analyses were performed in the individual samples, while other parts were conducted using the combined data (for the sake of increasing statistical power). In each of the following sections, we state which data set was used.

### Measures of Self-Complexity

We started with examining psychometric properties of alternative measures of self-complexity: the dimensionality statistic (H), quantity of self-aspects (NSA) and overlap among them (OL), and a composite index of these two components of self-complexity (SC). The subsequent analyses were based on combined samples for which the appropriate scores were available (i.e., Study 1b and Study 1c samples).

To test the internal consistency of each of the self-complexity measure, split-half reliability coefficients were calculated, following the procedure of Rafaeli-Mor et al. (1999). Instead of using a participant’s full trait sorting, the relevant measures (NSA, OL, H, and SC) were computed separately on two subsets of traits: the 30 odd-numbered traits and the 30 even-numbered traits. Scores on each measure within one subset of traits were then correlated with the respective scores within the other half of the traits. The resulting split-half correlations were corrected by the Spearman-Brown formula to obtain split-half reliability estimates. The split-half reliability estimate of NSA was the highest (*r* = 0.93), followed by Linville’s H statistic (*r* = 0.85), OL (*r* = 0.69), and Sakaki’s SC statistic (*r* = 0.57). Overall, the split-half reliability coefficients were moderate or satisfactory, with the number of self-aspects and the H statistic showing greater reliability. The reliability estimates for Linville’s H measure and NSA matched the values found by Rafaeli-Mor et al. (1999) in the semi-random splitting. The reliability coefficient for OL was higher than that reported by Rafaeli-Mor et al. (1999) for the valenced split, but remained below the coefficients that were computed in the semi-random splitting.

The participants used an average of 15.44 (*SD* =6.53) trait adjectives in their self-descriptions. The number of self-aspects (NSA) in the present study ranged from 1 to 12, with a mean of 4.90 (*SD* =1.93), overlap (OL) ranged from 0.00 to 1.00 with a mean of 0.17 (*SD* =0.15),^4^ the H statistic ranged from 0.12 to 3.77 with a mean of 1.51 (*SD* =0.61), and the SC statistic ranged from 3.08 to 1200.00 with a mean of 45.31 (*SD* =71.17). All data were checked for normality. The skewness of the variables ranged from 0.61 to 9.56, and their kurtosis ranged from 0.61 to 136.09. Only the SC scores showed a severe departure from normality based on Kline’s (1998) rule (i.e., skew index absolute value <3; kurtosis index absolute values <10). There were significant gender differences in the number of traits used, the number of self-aspects, overlap, and Linville’s H statistic. Women (1) used more traits to describe themselves (*M*_women_ =16.61 [*SD* =6.01] vs. *M*_men_ =13.78 [*SD* =6.89]), *U* =36987.00, *Z* = −6.14, *p* < 0.001, *r* = 0.24, (2) identified more self-aspects (*M*_women_ =5.13 [*SD* =1.81] vs. *M*_men_ =4.58 [*SD* =2.06]), *U* =41133.50, *Z* = −4.47, *p* < 0.001, *r* = 0.17, (3) had more interrelated self-aspects (*M*_women_ =0.19 [*SD* =0.15] vs. *M*_men_ =0.15 [*SD* =0.16]), *U* =40603.00, *Z* = −4.25, *p* < 0.001, *r* = 0.17, and (4) had higher H scores (*M*_women_ =1.62 [*SD* =0.57] vs. *M*_men_ =1.34 [*SD* =0.63]), *U* =36271.50, *Z* = −6.44, *p* < 0.001, *r* = 0.25, than did men.

The simple correlation analysis revealed that the H statistic had a positive association with the number of self-aspects (*r* = 0.59, *p* < 0.001, *r*^*2*^ = 0.35), and thus appeared to reflect this element of self-complexity quite well. This is consistent with the findings of Linville (1987) and others (e.g., Brown and Rafaeli 2007; Rafaeli-Mor et al. 1999). However, contrary to Linville’s expectation that the H statistic will reflect high distinctiveness among roles, the H statistic and overlap were positively related in this sample (*r* = 0.27, *p* < 0.001, *r*^*2*^ = 0.08). Similar result was previously reported by Constantino et al. (2006), Rafaeli-Mor et al. (1999), and Luo et al. (2009). We examined the scatterplot of the two variables and used regression analysis to test the significance of a quadratic effect for overlap. The analysis indicated that both linear and nonlinear effects were significant (β =0.15 and β = −0.13, *p* < 0.001, respectively), suggesting a concave down quadratic trend in the relationship between overlap and the H scores. This observation was even more important since the mean level of overlap in our sample was very low (as was in Rafaeli-Mor et al.’s study, *M* =0.13, Constantino et al.’s study, *M* =0.17, and Luo and Watkins’ study, *M* =0.18) and lied in the 1st theoretical quartile. A high percentage of 96 % of the participants had overlap below, and merely 4 %, above, the theoretical midpoint of 0.50. We used the theoretical midpoint of 0.50 to separate high-OL and low-OL groups, and then performed regression analysis for each group (controlling for the effect of the number of self-aspects and the number of attributes). As predicted, the H statistic was positively predicted by overlap in the low-OL group (β =0.10, *p* < 0.001), but negatively predicted by overlap in the high-OL group (β = −0.24, *p* < 0.001). These findings suggest that there were certain circumstances in which greater overlap increased the value of H and other circumstances in which greater overlap reduced its value. More precisely, the relationship between Linville’s H measure and overlap followed an inverted U-shaped function, but because most overlap values were relatively small, the general relationship between them was positive.

Consistent with the findings of Linville (1987), the SC statistic was found to be positively related to the number of self-aspects (*r* = 0.36, *p* < 0.001, *r*^*2*^ = 0.13) and negatively associated with overlap (*r* = −0.41, *p* < 0.001, *r*^*2*^ = 0.17). In agreement with Brown and Rafaeli (2007), the two component measures of self-complexity (the number of roles and overlap) were unrelated (*r* = 0.04, *ns*). But controversy emerged as the H statistic and the SC statistic turned out to be unrelated (*r* = 0.05, *ns*). Since both measures theoretically capture the same construct, this can be only explained by the difference in calculation formula.

Additional analysis showed that the H statistic is problematic for another reason. It was found to be strongly and positively linked to the number of trait adjectives used in the person’s sort (*r* = 0.97, *p* < 0.001, *r*^*2*^ = 0.94). And this is not without importance, since participants used positive self-descriptors much more frequently than negative ones (*χ*^*2*^(1) =6.25, *p* = 0.012). This tendency reflects a form of self-enhancement (e.g., Sedikides 1993). Expectedly, the H statistic showed a stronger correlation with the number of positive trait adjectives (*r* = 0.90, *p* < 0.001, *r*^*2*^ = 0.81) than with the number of negative trait adjectives (*r* = 0.56, *p* < 0.001, *r*^*2*^ = 0.32). The difference between the correlation coefficients was significant (*z* = 14.88, *p* < 0.001). This turned out to be true for NSA (*r* = 0.44 and *r* = 0.25, *z* = 3.96, *p* < 0.001) and OL (*r* = 0.25 and *r* = 0.05, *z* = 3.71, *p* < 0.001) as well. On the other hand, the same correlation analysis performed for the SC statistic revealed that neither the total number of chosen adjectives nor the number of positive or negative adjectives were related to the SC statistic (*r* = 0.04, *r* = 0.02, and *r* = 0.05, *ns*, respectively).^5^

To ascertain these relations and determine the most important predictors of both Linville’s H and Sakaki’s SC statistic, simultaneous multiple regression analyses were conducted with the number of self-aspects, overlap, and the number of chosen adjectives as predictors and either the H statistic or the SC statistic as the dependent variable. Squared semipartial correlations were calculated to estimate the unique contribution of each predictor to the variance in self-complexity scores. We also tested for multicollinearity using a rule of thumb associated with the variance inflation factor (VIF <5).

As can be seen in Table 2, NAS, OL, and NAT were significant unique predictors of self-complexity, regardless the measure that was used. The number of chosen adjectives emerged as the strongest (and positive) predictor of Linville’s H statistic (β = 0.87, *p* < 0.001). Positive associations between the H statistic and both number of roles and overlap held even when controlling for all the other predictor variables (β = 0.19, *p* < 0.001 and β = 0.06, *p* < 0.001, respectively). The number of self-aspects was the strongest (and positive) predictor (β = 0.42, *p* < 0.001) of Sakaki’s SC statistic, closely followed by overlap (β = −0.40, *p* < 0.001), which remained negatively associated with the SC statistic. The regression model (*F*(3, 642) =5765.86, *p* < 0.001) accounted for 96 % of the variance in the H score, with NAT explaining 57.5 % of the variance, NSA approximately 3 %, and OL an additional 0.3 %, as reflected by the squared semipartial correlation. Thus both NSA and OL played only a minor role in self-complexity indicated by the H statistic. For Sakaki’s SC statistic, the regression model accounted for 31 % of the variance (*F*(3, 530) =77.80, *p* < 0.001), with OL and NSA explaining 15.5 % and 13 % of the variance, respectively. The remaining 1.6 % was attributable to NTA.^6^

**Table 2:** Summary of multiple regression analysis with either the H or SC statistic as the dependent variable

	Linville’s H statistic		Sakaki’s SC statistic	
Variable	β	*t*	β	*t*
NSA	0.19	22.76***	0.42	10.01***
OL	0.06	7.84***	−0.40	−10.90***
NAT	0.87	101.46***	−0.14	−3.46***
Model	*R* ^*2*^ = 0.96, *F*(3, 642) =5765.86, *p* < 0.001		*R* ^*2*^ = 0.31, *F*(3, 530) =77.80, *p* < 0.001	

*N* = 652

*NSA* number of self-aspects, *OL* overlap, *NAT* number of trait adjectives

*** *p* <0.001

The above results suggest that the SC statistic serves as a more adequate measure of the self-complexity as conceptualized by Linville (1987). However, since the equation for computing the SC statistic may cause a divide by zero error, using the two-component approach and analyzing NSA and OL separately seems to be most suitable. It is also worth noting that Linville’s H statistic, reflecting predominantly the quantity of self-descriptions, constitutes the measure of differentiation, and not self-complexity, according to Zajonc’s (1960) original taxonomy.

### Measures of Self-Concept Differentiation

The following analyses aimed at providing validity information on the Self-Incoherence Scale and exploring the convergence among different measures designed to assess self-concept differentiation: the average standard deviation of trait ratings across roles (SCD_SD_),the average correlation among the roles (SCD_R_), and the proportion of unshared variance among the roles (SCD_VAR_). The analyses in this section were based on combined samples for which the appropriate scores were available (i.e., Study 1a and Study 1b samples).

Internal consistency reliability of the scale was examined. The standardized Cronbach’s alpha coefficient of the composite index, based on the intercorrelations among trait-specific standard deviations, was 0.83. The mean corrected item-total correlation for the 7 traits was *r* = 0.57, indicating that those participants who were variable on one trait tended to be variable on the others. Although the reliability coefficient was slightly lower than that of the original study of Styła et al. (2010, α =0.90), it was nevertheless acceptable.

The self-differentiation scores calculated from an average standard deviation (SCD_SD_) in the present study ranged from 0.00 to 3.03, with a mean of 0.98 (*SD* =0.36), the index based on an average correlation (SCD_R_) ranged from 0.10 to 1.00, with a mean of 0.42 (*SD* =0.13), and the index derived from factor analysis (SCD_VAR_) ranged from 0.00 to 67.92, with a mean of 45.77 (*SD* =11.07).^7^ For all three variables, skewness and kurtosis were within acceptable limits (skewness: −0.77 to 1.18; kurtosis: 0.75 to 3.67). No significant gender differences were observed.

The zero-order correlation revealed that the SCD index based on an average standard deviation was only weakly correlated with Campbell et al.’s (2003) index (*r* = −0.27, *p* < 0.001, *r*^*2*^ = 0.07) and Block’s (1961) index (*r* = 0.27, *p* < 0.001, *r*^*2*^ = 0.07). These findings are contrary to those by Donahue et al. (1993) who reported high degree of convergence between alternative indexes. In accordance with expectations, Campbell et al.’s (2003) and Block’s (1961) indexes were strongly associated (*r* = −0.95, *p* < 0.001, *r*^*2*^ = 0.90).

Additional concerns raised with respect to the fact that all personality traits included in the Self-Incoherence Scale were positively valenced. Thus, it is possible that the SCD score represents the consistency of endorsing desirable traits rather than self-concept differentiation, as conceptualized in the literature (Donahue et al. 1993). To test this hypothesis, we compared the SCD scores in two groups: participants who rated themselves highly on the 7 positive traits and those, who claimed they lacked them. For each participant, we averaged their ratings on each trait across the five roles. The compared groups were composed of participants who obtained extremely low and extremely high scores on a given trait. A standard deviation criterion was used as cut-off point. The Mann–Whitney U tests showed there were significant differences in trait variability scores between the two groups on every personality trait analyzed (effect size range *r* = 0.47 to 0.78, average *r* = 0.58). Specifically, participants who rated a positive personality trait as highly descriptive of themselves received lower standard deviation scores for that trait_,_ indicating a more stable self-concept (see Table 3). Similar results were obtained in correlation analysis of mean trait rating with cross-role standard deviation for the respective trait. The correlation coefficients ranged from *r* = −0.32 to *r* = −0.54, with the average coefficient being approximately −0.39 (*p* < 0.001, range of *r*^*2*^ = 0.10 to 0.29, average *r*^*2*^ = 0.16).

**Table 3:** Trait variability in individuals with high and low ratings on traits included in the Self-Incoherence Scale

	Low ratings	High ratings			
Variable	*M* (*SD*)	*M* (*SD*)	*U*	Z	Effect size *r*
Trait 1	1.35 (0.64)	0.77 (0.36)	4093.50***	−7.65	0.47
Trait 2	1.33 (0.62)	0.57 (0.35)	2711.50***	−10.51	0.63
Trait 3	1.27 (0.68)	0.22 (0.23)	707.50***	−12.21	0.78
Trait 4	1.21 (0.69)	0.57 (0.36)	3179.00***	−7.49	0.48
Trait 5	0.99 (0.57)	0.39 (0.27)	1600.00***	−9.59	0.65
Trait 6	1.17 (0.65)	0.61 (0.38)	3712.50***	−7.667	0.48
Trait 7	1.25 (0.65)	0.50 (0.42)	3537.50***	−9.606	0.57

*N* = 868

*** *p* < 0.001

We also performed Mann–Whitney U tests to compare all three SCD indexes between participants who generally viewed themselves positively and those who evaluated themselves more negatively. The two groups were obtained simply by averaging participants’ ratings for all 7 personality traits. A standard deviation criterion was used as cut-off point. Once again, as shown in Table 4, self-differentiation index based on an average standard deviation was found to be associated with a tendency to ascribe desirable traits to the self (*U* =3025.50, *p* < 0.001, *r* = 0.57).^8^ The Pearson correlation coefficient of these two variables was *r* = −0.40 and was statistically significant (*p* < 0.001, *r*^*2*^ = 0,16). This effect, however, was not observed for the other two SCD indexes.

**Table 4:** Self-concept differentiation difference between individuals with high and low mean ratings

	Low ratings	High ratings			
Variable	*M* (*SD*)	*M* (*SD*)	*U*	Z	Effect size *r*
SCD_SD_	1.13 (0.45)	0.69 (0.24)	3025.50***	−9.33	0.57
SCD_R_	0.44 (0.14)	0.44 (0.13)	6268.50	−0.27	0.02
SCD_VAR_	45.21 (12.57)	43.89 (11.57)	5909.00	−0.99	0.07

*N* = 868

*SCD*_*SD*_ average standard deviation of trait ratings across roles, *SCD*_*R*_ average correlation among the roles, *SCD*_*VAR*_ proportion of unshared variance among the roles

*** *p* < 0.001

The above results suggest that there is varying correspondence between alternative ways of measuring self-concept differentiation, and, more importantly, that self-concept differentiation – operationalized as the average standard deviation – may not be independent of the contents of the self-concept (see also Locke 2006).

### Relationship Between Self-Complexity and Self-Concept Differentiation

We examined whether measures of self-concept structure, reflecting differentiation and integration (unity), were related to one another. These analyses were based on Study 1b sample data.

Correlation analysis (see Table 5) between self-complexity, its components and self-concept differentiation revealed a weak, negative association between overlap and SCD index based on an average standard deviation (*r* = −0.13, *p* = 0.004, *r*^*2*^ = 0.02). Considering the dependence of SCD_SD_ on mean trait rating (cf. Tables 3 and 4), we decided to examine this result further. The zero-order correlation showed that along with OL and SCD_SD_ being correlated to each other, both were significantly correlated to the mean trait rating (*r* = 0.12, *p* = 0.005 and *r* = −0.40, *p* < 0.001, respectively). To test for the possible confounding effect of the mean trait rating, a hierarchical regression was used in which the SCD_SD_ score was regressed on overlap (Step 1), and participants’ mean rating for personality traits (Step 2). After entering the mean trait rating in Step 2, the model explained 16.5 % of the variance in the SCD_SD_ score (Δ*R*^*2*^ = 0.15, *F*(1, 515) =92.23, *p* < 0.001). The mean trait rating emerged as a significant predictor (β = −0.39, *p* < 0.001), whereas the effect of overlap was no longer significant (β = −0.08, *ns*).^9^

**Table 5:** Correlation matrix of measures of self-complexity and self concept differentiation

Variable	OL	H	SC	SCD_SD_	SCD_R_	SCD_VAR_
NSA	0.10*	0.56***	0.29***	−0.03	−0.01	0.02
OL	–	0.33***	−0.54***	−0.13**	0.04	−0.04
H		–	0.03	−0.08	0.03	0.00
SC			–	0.09	−0.10	0.08
SCD_SD_				–	−0.28***	0.28***
SCD_R_					–	−0.95***

*N* = 521

*NSA* number of self-aspects, *OL* overlap, *H* Linville’s self-complexity index, *SC* Sakaki’s self-complexity index, *SCD*_*SD*_ average standard deviation of trait ratings across roles, *SCD*_*R*_ average correlation among the roles, *SCD*_*VAR*_ proportion of unshared variance among the roles

*** *p* < 0.001, ** *p* < 0.01, * *p* < 0.05

No further associations between measures of self-complexity and measures of self concept differentiation were found, suggesting that they are, to a large extent, independent constructs. A similar conclusion was drawn by Campbell et al. (2003), and Lutz and Ross (2003).

### Identity Correlates of Self-Complexity and Self-Concept Differentiation

Traditionally, a stable and coherent personal identity is considered to be essential for psychological health and adaptive functioning. According to Erikson (1980), the subjective experience of personal identity actually gives rise to a preconscious sense of personal well-being. We examined the self structure–identity relationship, operationalizing identity through measures of identity processes and identity senses.

Basic statistical description of identity variables in combined sample is presented in Table 6. The levels of skewness and kurtosis exhibited by our data were below those that Kline (1998) specifies as problematic (skewness: −0.40 to 0.25; kurtosis: −0.25 to 0.26). An analysis of gender differences by means of Mann–Whitney U test indicated that men had a higher sense of inner contents (*U* =107833.50, *Z* = −2.47, *p* = 0.013, *r* =0.08), uniqueness (*U* =100746.00, *Z* = −4.00, *p* < 0.001, *r* = 0.13), their own boundaries (*U* =96659.00, *Z* = −4.96, *p* < 0.001, *r* = 0.16), and self-worth (*U* =94079.00, *Z* = −4.76, *p* < 0.001, *r* = 0.15), than women. Moreover, men, as compared to women, had higher scores on overall sense of identity (*U* =99912.50, *Z* = −4.27, *p* < 0.001, *r* = 0.14). With respect to identity processes, women endorsed identity accommodation more than men (*U* =1098.00, *Z* = −4.30, *p* < 0.001, *r* = 0.03), and men scored higher on identity balance than women (*U* =1496.00, *Z* = −2.23, *p* = 0.026, *r* = 0.02).

**Table 6:** Descriptive statistics and gender differences for identity senses and identity processes

Variable	*M*_total_ (*SD*)	*M*_women_ (*SD*)	*M*_men_ (*SD*)	*U*	Z	Effect size *r*
SIC	2.18 (0.48)	2.15 (0.46)	2.22 (0.51)	107,833.50*	−2.47	0.08
SU	1.71 (0.48)	1.66 (0.49)	1.78 (0.47)	100,746.00***	−4.00	0.13
SOB	1.50 (0.45)	1.44 (0.44)	1.60 (0.44)	96,659.00***	−4.96	0.16
SC	1.95 (0.46)	1.93 (0.44)	1.97 (0.49)	112,046.00	−1.55	0.05
SCT	1.90 (0.41)	1.88 (0.40)	1.92 (0.44)	113,717.00	−1.14	0.04
SSW	1.95 (0.49)	1.89 (0.48)	2.04 (0.49)	94,079.00***	−4.76	0.15
GSI	1.86 (0.33)	1.82 (0.31)	1.92 (0.34)	99,912.50***	−4.27	0.14
IAS	3.59 (0.87)	3.63 (0.91)	3.58 (0.84)	1943.00	−0.16	0.00
IAC	3.79 (1.08)	4.20 (1.05)	3.34 (0.94)	1098.00***	−4.30	0.03
IBL	4.90 (0.93)	4.74 (0.80)	5.08 (1.05)	1496.00*	−2.23	0.02

Descriptive statistics for identity senses are based on 1001 participants; descriptive statistics for identity processes are based on 127 participants

*SIC* sense of having inner contents, *SU* sense of uniqueness, *SOB* sense of one’s own boundaries, *SC* sense of coherence, *SCT* sense of continuity over time, *SSW* sense of self-worth, *GSI* global sense of identity, *IAS* assimilation, *IAC* accommodation, *IBL* balance

*** *p* < 0.001, * *p* < 0.05

Table 7 contains the zero-order correlations between self-complexity, self concept differentiation, and identity measures. All observed correlations were rather weak (*r* ≤0.34) and seemingly inconsistent. Of all self-complexity measures, relatively the strongest correlation emerged between the H statistic and identity balance (Study 1c: *r* = 0.31, *p* < 0.001, *r*^*2*^ = 0.10). Linville’s measure was also related to most of the identity senses (average *r* = 0.11, average *r*^*2*^ = 0.02) and global sense of identity (average *r* = 0.16, average *r*^*2*^ = 0.03). These correlations were all positive with the exception of the one with sense of one’s own boundaries. Overlap showed significant associations with some of the identity senses (average *r* = 0.02, average *r*^*2*^ = 0.01) and global sense of identity (average *r* = 0.02, average *r*^*2*^ = 0.01). Though the direction and significance of these associations was not consistent across the two studies. The only significant correlation of the number of self-aspects was a negative one with sense of one’s own boundaries (Study 1b: *r* = −0.12, *r*^*2*^ = 0.01), and the only significant correlation of Sakaki’s SC statistic was a negative one with identity accommodation (Study 1c: *r* = −0.21, *r*^*2*^ = 0.05). Negative associations emerged between SCD_SD_ and most of the identity senses (average *r* = −0.18, average *r*^*2*^ = 0.05) and global sense of identity (average *r* = −0.24, average *r*^*2*^ = 0.07). Clearly weaker correlations were found between identity senses and SCD_R_ (average *r* = 0.03, average *r*^*2*^ = 0.004) and SCD_VAR_ (average *r* = −0.04, average *r*^*2*^ = 0.005). Both of these self-concept differentiation indices were uncorrelated with the global sense of identity score (on average, *r* = 0.04, *r*^*2*^ = 0.002 and *r* = −0.06, *r*^*2*^ = 0.004, respectively).

**Table 7:** Correlation matrix of measures of self-complexity, self-concept differentiation, and identity

Sample	SIC	SU	SOB	SC	SCT	SSW	GSI	IAS	IAC	IBL
NSA										
Study 1b	0.03	−0.02	−0.12**	0.01	0.04	0.06	0.00			
Study 1c	−0.01	0.06	0.07	−0.05	0.04	0.10	0.05	−0.01	−0.07	0.08
OL										
Study 1b	0.08	0.01	−0.05	0.09*	0.16***	0.09*	0.09*			
Study 1c	−0.09	−0.08	0.10	0.03	−0.07	−0.06	−0.04	−0.15	−0.08	0.06
H										
Study 1b	0.11*	0.06	−0.09*	0.06	0.11*	0.14***	0.09*			
Study 1c	0.14	0.22*	0.15	0.06	0.17	0.23*	0.23*	0.10	−0.05	0.31***
SC										
Study 1b	0.01	−0.06	−0.02	−0.01	−0.06	0.00	−0.03			
Study 1c	0.13	0.02	−0.12	0.06	0.04	0.11	0.06	−0.04	−0.21*	0.06
SCD_SD_										
Study 1a	−0.34***	0.01	−0.13*	−0.39***	−0.34***	−0.31***	−0.34***			
Study 1b	−0.18***	−0.02	−0.01	−0.19***	−0.11*	−0.12**	−0.15***			
SCD_R_										
Study 1a	0.06	0.00	−0.04	0.13*	0.11*	0.02	0.06			
Study 1b	−0.01	−0.05	0.04	0.05	0.06	−0.02	0.01			
SCD_VAR_										
Study 1a	−0.09	0.01	0.02	−0.14*	−0.13*	−0.05	−0.08			
Study 1b	−0.01	0.04	−0.06	−0.07	−0.06	0.01	−0.03			

*NSA* number of self-aspects, *OL* overlap, *H* Linville’s self-complexity index, *SC* Sakaki’s self-complexity index, *SCD*_*SD*_ average standard deviation of trait ratings across roles, *SCD*_*R*_ average correlation among the roles, *SCD*_*VAR*_ proportion of unshared variance among the roles, *SIC* sense of having inner contents, *SU* sense of uniqueness, *SOB* sense of one’s own boundaries, *SC* sense of coherence, *SCT* sense of continuity over time, *SSW* sense of self-worth, *GSI* global sense of identity, *IAS* assimilation, *IAC* accommodation, *IBL* balance

*** *p* ≤ 0.001, ** *p* ≤ 0.01* *p* ≤ 0.05

To ascertain the extent to which each of the self complexity and self-concept differentiation measures exhibited associations with identity variables that were independent of their associations with other variables, regression analyses were conducted. Identity scores were regressed on self-structure variables that were available in the data set. Due to the fact that specific identity senses were correlated with one another (average *r* = 0.41, *p* < 0.001), we only used the global sense of identity scores. Also, there were a number of cases for which the SC scores and scores on the two SCD indices based on the cross-role correlation matrix could not be obtained because of calculation problems (i.e., a division by zero error and an undefined correlation with zero variance). Since the missing cases may have caused the samples to be biased, we excluded these measures from the regression analyses described below.^10^ Lastly, since some of the self-structural measures have been shown to be sensitive to variations in self-contents variables (e.g., the proportion of positive traits adjectives used in the sort and the mean trait rating), we performed regressions including them as predictors to control for their respective effects.

In all models, multicollinearity was examined to determine if any of the independent or control variables were a significant function of each other. As expected, since the H score and the number of traits were very highly correlated, the tolerances for these variables were very low and the VIFs exceed the value of 10. In order to avoid the multicollinearity problem, only the number of traits has been included in the analysis. The tolerance and the variance inflation factors in the final regression analyses were well within the acceptable range (tolerance >0.20, VIF <5).

In general, control variables (i.e., related to richness and favorability of self-depiction) were more consistent predictors of personal identity than variables related to self-concept structure (see Table 8).^11^ Individuals with a strong sense of identity and who were identity balanced were more likely to have very rich self-defining aspects and to describe themselves with more positive attributes.

**Table 8:** Summary of beta weights for regression analyses with identity measures as the dependent variable

	GSI			IAS	IAC	IBL
Source	Study 1a	Study 1b	Study 1c	Study 1c	Study 1c	Study 1c
NSA		−0.07	−0.11	−0.11	−0.12	−0.10
OL		0.00	−0.12	−0.19*	−0.07	−0.03
SCD_SD_	−0.22***	−0.03				
NAT		0.11*	0.39***	0.19	−0.05	0.44***
PPAT		−0.01	0.29***	0.12	−0.22*	0.31***
MTR	0.30***	0.33***				
Model	*R* ^*2*^ = 0.19, *F*(2, 330) =39.00, *p* < 0.001	*R* ^*2*^ = 0.14, *F*(6, 504) =13.25, *p* < 0.001	*R* ^*2*^ = 0.17, *F*(4, 114) =5.84, *p* < 0.001	*R* ^*2*^ = 0.06, *F*(4, 111) =1.68, *ns*	*R* ^*2*^ = 0.07, *F*(4, 111) =2.01, *ns*	*R* ^*2*^ = 0.21, *F*(4, 110) =7.53, *p* < 0.001

*NSA* number of self-aspects, *OL* overlap, *SCD*_*SD*_ average standard deviation of trait ratings across roles, *NAT* number of trait adjectives, *PPAT* proportion of positive trait adjectives, *MTR* mean trait rating, *GSI* global sense of identity, *IAS* assimilation, *IAC* accommodation, *IBL* balance

*** *p* ≤ 0.001,** *p* < 0.01, * *p* < 0.05

With this in mind, it is possible to interpret psychologically the obtained results. The global sense of identity was negatively predicted by SCD_SD_ (Study 1a: β = −0.22, *p* < 0.001), showing that a differentiated self-concept was associated with a weakening of a sense of identity. Approximately 4 % of the GSI variance was explained by SCD_SD_. OL emerged as a significant predictor of IAS (Study 1c: β = −0.19, *p* = 0.046), suggesting that a unified self-structure was related to lesser use of assimilation, probably since there was hardly any discrepancy that needed to be resolved. About 3.5 % of the variance in the IAS scores was attributable to OL.

Overall, these correlation and regression findings would support the assumption that a sense of identity is related to coherence across self-aspects rather than their complexity and diversity. At the same time, they suggest that certain relationships of structural variables with well-being outcomes could be explained by content-based variables.

### Cognitive Correlates of Self-Complexity and Self-Concept Differentiation

Drawing on the ideas of positive psychology, we posit that psychological well-being includes the development of various reflective and self-reflective capacities. Therefore, in this section, we address the issue of adaptive significance of structural aspects of self by exploring their relationships with need for cognition, reflection, and integrative self-knowledge. The following analyses were based on Study 1b sample data.

In our sample, the scores on need for cognition ranged from 56.00 to 175.00 (*M* =127.39, *SD* =17.92), on reflection ranged from 1.00 to 5.00 (*M* =3.26, *SD* =0.77), and for integrative self-knowledge ranged from 0.00 to 4.00 (*M* =2.36, *SD* =0.63). For the current data set, the skewness of the variables ranged from −0.18 to 0.09, and the kurtosis ranged from −0.16 to 0.38. These estimates did not identify any serious violations of normality. To examine gender differences, for each variable, an independent Mann–Whitney U test was computed comparing the male and female scores. Women (*M* =3.21, *SD* =0.76) scored significantly lower than men (*M* =3.34, *SD* =0.77) on reflection (*U* =31578.00, *Z* = −2.12, *p* = 0.034, *r* = 0.09). In terms of other variables, no significant gender differences were observed.

The observed correlations between measures of self-complexity and self-concept differentiation, and thinking dispositions are given in Table 9. Need for cognition correlated with all three indices of self-concept differentiation. The strongest association was observed between NCS and SCD_SD_ (*r* = −0.29, *p* < 0.001, *r*^*2*^ = 0.09). Reflection correlated with the number of self-aspects, Linville’s H statistic, and self-concept differentiation index calculated from an average standard deviation. The latter association was the strongest (*r* = −0.18, *p* < 0.001, *r*^*2*^ = 0.03). Finally, integrative self-knowledge correlated with overlap, the H and the SC statistics, and self-concept differentiation index based on an average standard deviation. The association of greatest magnitude was between ISK and OL (*r* = 0.21, *p* < 0.001, *r*^*2*^ = 0.04). It should be pointed out that all of the correlation coefficients were rather small, even when statistically significant; the largest explained about 9 % of the variance.

**Table 9:** Correlation matrix of measures of self-complexity, self-concept differentiation, and thinking dispositions

Variable	NCS	RQ	ISK
NSA	0.02	0.11*	0.00
OL	0.08	0.06	0.21***
H	0.07	0.12**	0.15***
SC	0.00	0.06	−0.11*
SCD_SD_	−0.29***	−0.18***	−0.18***
SCD_R_	0.10*	0.06	0.07
SCD_VAR_	−0.11*	−0.05	−0.07

*NSA* number of self-aspects, *OL* overlap, *H* Linville’s self-complexity index, *SC* Sakaki’s self-complexity index, *SCD*_*SD*_ average standard deviation of trait ratings across roles, *SCD*_*R*_ average correlation among the roles, *SCD*_*VAR*_ proportion of unshared variance among the roles, *NCS* need for cognition, *RQ* reflection, *ISK* integrative self-knowledge

*** *p* ≤ 0.001, ** *p* < 0.01* *p* < 0.05

Although the zero-order correlations are instructive, they may be misleading. Therefore, a subsequent series of regression analyses was performed to examine the independent effects of measure of self-complexity and self-concept differentiation on each cognitive variable. The rationale guiding inclusion of the predictor variables was similar to the analyses in the previous section. Sakaki’s SC measure, the SCD_VAR_ index, and the SCD_R_ index were excluded to avoid bias in the sample and to retain sample size.^12^ Linville’s H measure was excluded to avoid multicollinearity. Three variables related to richness and favorability of self-concept were included in the models to control for their potential effects. The beta weights (standardized regression coefficients) are reported in Table 10.

**Table 10:** Summary of beta weights for regression analyses with thinking dispositions as the dependent variable

Source	NCS	RQ	ISK
NSA	−0.01	0.07	−0.08
OL	0.01	−0.01	0.16***
SCD_SD_	−0.19***	−0.16***	−0.11*
NAT	0.05	0.11*	0.13**
PPAT	0.05	0.10*	−0.01
MTR	0.22***	−0.02	0.15**
Model	*R* ^*2*^ = 0.14, *F*(6, 510) =13.57, *p* < 0.001	*R* ^*2*^ = 0.06, *F*(6, 510) =5.60, *p* < 0.001	*R* ^*2*^ = 0.11, *F*(6, 509) =10.81, *p* < 0.001

*NSA* number of self-aspects, *OL* overlap, *SCD*_*SD*_ average standard deviation of trait ratings across roles, *NAT* number of trait adjectives, *PPAT* proportion of positive trait adjectives, *MTR* mean trait rating, *NCS* need for cognition, *RQ* reflection, *ISK* integrative self-knowledge

*** *p* < 0.001, ** *p* < 0.01, * *p* < 0.05

The obtained results indicated, again, that the control variables were more strongly related to thinking dispositions than were the main predictors. Individuals who gave more elaborated self-descriptions and expressed more favorable self-evaluations used adaptive cognitive thinking to a greater degree. Of the included structural features of the self-concept, self-concept differentiation emerged as a significant predictor of need for cognition (β = −0.19, *p* < 0.001) as well as reflection (β = −0.16, *p* ≤0.001), and integrative self-knowledge (β = −0.11, *p* = 0.016), accounting for, respectively, 3 %, 2 %, and 1 % of the criterion variance. These effects suggested that active cognitive processing accompanied less differentiated self-structure. Overlap was independently associated with integrative self-knowledge (β =0.16, *p* < 0.001), showing that a tendency to integrate past, present, and desired future self-experience into a meaningful whole was directly linked to a unified self-structure. The predictive value of overlap for integrative self-knowledge was approximately 2.4 %.^13^

Altogether, the obtained results indicated that the thinking dispositions of interest were not substantially predicted from the included structural features of the self-concept. Although relatively small in magnitude, these effects suggested that engaging in functionally adaptive cognitive processing was associated with a more coherent self-concept.

## General Discussion

Individual differences in the self-concept structure have been a topic of considerable interest for at least three decades and have been debated as to their validity and adaptive significance. Thus, in this paper, we tackled issues related to the validity of operationalization and adaptive value of self-complexity and self-concept differentiation. The results of the present investigation can be summed up as follows:

1. *The obtained results suggest that both singular indices of self-complexity – Linville’s H statistic and Sakaki’s SC statistic – are lacking, although for different reasons.*

Correlation and regression analyses showed that the H score was positively associated with the number of self-aspects as well as with the overlap among them. Thus, in contrast to Linville’s (1985, 1987) prediction, not only numerous self-aspects but also more role overlap strengthen one’s H score. The effect of overlap proved particularly inconsistent across different studies, ranging from positive (e.g., Constantino et al. 2006; Luo and Watkins 2009; Rafaeli-Mor et al. 1999) to even nonsignificant (e.g., Brown and Rafaeli 2007; Engįn 2004; Heath 2011; Rafaeli-Mor and Steinberg 2002), with no consensus on its association to Linville’s H measure. These potentially conflicting findings could all be correct. Inspection of our data indicated that the relationship between the H statistic and overlap was, in fact, inverted U-shaped (see also Luo and Watkins 2008, 2009; Luo et al. 2009). That is, an increase in overlap initially increases the H score, but when overlap exceeds a certain threshold, the H score decreases. Thus, depending on range of overlap values observed, the H statistic function takes on a different shape. The most possible explanation for our overall positive result is that the range of overlap in our data was sufficiently low, so that we did not observe the decrease in the H score – only the increase. The same explanation could account for previous failures (e.g., Constantino et al. 2006; Luo and Watkins 2009; Rafaeli-Mor et al. 1999) to identify the theoretically assumed negative effect of overlap on Linville’s H measure.

Moreover, the H statistic turned out to be influenced by the number of traits used in self-definition. In fact, the total number of traits used in the trait-sort task appeared to be the most important predictive factor for the H score, and a significant mediator in the NSA–H and OL–H relationships. Taken together, these data indicate that Linville’s H statistic, at best, could be considered an indirect measure of role quantity or simply a measure of the number of traits endorsed (see also Zajonc 1960). Corresponding conclusions were drawn by Locke (2003), Rafaeli-Mor et al. (1999), and Solomon and Haaga (2003). In addition, since the H statistic is highly sensitive to the number of traits, and people tend to ascribe far more positive traits than negative traits to the self, the H statistic will somewhat reflect self-esteem. This was previously recognized by Woolfolk et al. (1995) and Campbell et al. (2003), who found that self-complexity was influenced by the evaluative composition of the attributes sorted (i.e., the ratio of positive to negative attributes). Our analyses revealed that both component measures of self-complexity were also affected by the number of traits used, although to a much lesser extent than the H statistic. In addition, overlap was also found to be dependent on the favorability of the self-concept. This agrees with the results reported by Campbell et al. (2003) for the average correlation among self-aspects. One important question which we should address at this point is, whether we should interpret the obtained results as an indication of a positive self-complexity–self-esteem relationship or as a result of the method of calculation. While not entirely conclusive, our data, and those cited above, point to the second conclusion.

As for Sakaki’s SC statistic, it was found to be related positively to the number of self-aspects utilized and negatively to the overlap among these self-aspects, as Linville argued that it should. The amount of the SC statistic variance captured by the number of traits used in the sort was so small as to suggest that it probably was not meaningful. However, the mathematical formula of the SC statistic can potentially require division by zero and thus may yield uninterpretable results. Surprising as it may seem, neither Sakaki (2004, 2006) nor other authors that used this index (e.g., Borawski 2011) commented on this issue.^14^

Given the above and in agreement with Rafaeli-Mor and colleagues (1999), the two component measures of self-complexity, rather than singular measures, emerge as a more reasonable alternative in self-complexity assessment. Results showed that the component measures of self-complexity were uncorrelated with each other, thus providing support for their functional independence and for the two-dimensional nature of self-complexity, comprising both differentiation and a form of integration.

2. *The results found that measures of self-concept differentiation based on an average standard deviation of trait ratings across roles and derived from correlations between role identities were not convergent. Moreover, each of them share certain limitations.*

Although Campbell et al.’s (2003) and Block’s (1961) indices of self-concept differentiation were strongly intercorrelated, contrary to Donahue et al.’s (1993) finding, they had a weaker correlation with the measure recommended by Styła et al. (2010). We also found evidence for the non-convergence of these indices through an examination of their patterns of relationships with identity and cognitive variables. In particular, associations between indices derived from cross-role correlations and these other variables were considerably weaker and less consistent.

Furthermore, the SCD_SD_ index turned out to be biased, since participants rating positive traits as highly descriptive of themselves obtained considerably lower self-concept differentiation scores. Hence, it could be argued that the SCD_SD_ index reflects self-esteem (or self-esteem enhancement) in addition to self-concept differentiation. Similar concerns have been raised previously by Baird et al. (2006). Their studies provided evidence that the cross-role standard deviation conflate mean-level information with variability in trait expression. Baird et al. (2006) strongly suggested that this association is attributable to the constraints on the bivariate distribution and not to any underlying psychological process. They also pointed out that the relation of mean level and variability of traits will be more evident if the distribution of means is skewed. Because the Self-Incoherence Scale is restricted to positively valenced traits, and people generally view themselves positively, the means distribution in our data was skewed toward higher values (*Sk* = −0.49, *SES* =0.08). Thus, in regression analyses we controlled for trait mean levels and examined whether variability per se predicted well-being outcomes.

As to indices based on cross-role correlation matrix, they can only reflect the covariance rather than consistency between role-based personalities, meaning that they do not exactly reflect the theoretically defined construct of self-concept differentiation (Donahue et al. 1993). Moreover, as such they are sensitive to the within role variance, and thence any role identity that has no within variation has to be dropped from calculations or the indices remain undefined in such circumstances (see also Baird et al. 2006; Locke 2006). The chance of this occurring is lower when the number of self-descriptive traits is greater. However, with only seven self-descriptors, as in the Self-Incoherence Scale, this became an important issue. Regardless, this source of variance is not even relevant to what Donahue and colleagues (1993) have conceptualized as self-concept differentiation.

3. *The examination of relationships between measures of self-complexity and self-concept differentiation did not reveal any evident links between those seemingly similar constructs.*

Our overall results were comparable to those previously reported (e.g., Campbell et al. 2003; Constantino et al. 2006; Lutz and Ross 2003), as we found no unique associations between measures of self-complexity and self-concept differentiation. Correlation and regression analyses of self-complexity, its components, and self-concept differentiation on identity and cognitive variables yielded rather inconclusive results (see later in the text). Still, it seems safe to say that the patterns of associations for different structural features were neither similar, nor opposite. These findings support the postulate that complexity of the self-concept and self-concept differentiation are not the same phenomena. Moreover, since the two integration measures, namely overlap and self-concept differentiation, also appeared not to be uniquely related to one another, we might need to assume that they reflect different forms of integration. The above observations can also be understood in terms of the nature of the technique used. As noted in the introduction, the self-complexity task allows individuals to define their self-aspects in idiosyncratic ways, whereas in the self-concept differentiation task participants are constrained by limited and predefined roles. As pointed out by some authors (e.g., Constantino et al. 2006; Koch and Shepperd 2004; Lutz and Ross 2003), this distinction is important in that the former task may draw participants’ attention to more pleasant feelings of social specialization (a flexibility–rigidity dimension), whereas the latter task may promote focusing attention on rather unpleasant feelings of wearing many masks (an integration–fragmentation dimension).

4. *There were some direct associations of measures of self-complexity and self-concept differentiation with measures of identity and active cognitive processing. However, these associations were more limited than expected, and were influenced by the self-contents related variables.*

The focus of the current paper was to consider the relations between structural characteristics of the self-concept and two broad aspects of psychological adjustment: personal identity and thinking dispositions. The former was operationalized in terms of identity senses (Pilarska 2012, 2014a) and identity processes (Whitbourne et al. 2002). The latter were operationalized as individual differences in need for cognition (Cacioppo et al. 1996), reflection (Trapnell and Campbell 1999), and integrative self-knowledge (Ghorbani et al. 2008); all of which can be considered indicative of adaptive cognitive thinking.

As previously reported, several structural features of the self-concept appeared to be a function of the quantity and positivity of the self-descriptions. Not surprisingly, our results concerning the relationships of self-complexity and self-concept differentiation with adaptive outcomes were affected by self-contents related variables. Participants’ mean ratings of personality traits mediated the effects of self-concept differentiation on global sense of identity, need for cognition, and integrative self-knowledge. Also, the number of traits used in the sort mediated the effects of overlap on integrative self-knowledge. With this in mind, below we discuss the results after controlling for these variables.

Among the set of self-structure variables, self-concept differentiation showed negative association with global sense of personal identity, suggesting that describing oneself differently across social contexts was indicative of a weakened sense of identity. These results are in line with other reports suggesting that self-concept differentiation serves as a sign of psychological maladjustment (e.g., Campbell et al. 2003; Diehl and Hay 2010; Diehl et al. 2001; Donahue et al. 1993; Lutz and Ross 2003; McReynolds et al. 2000) and identity struggle (e.g., Block 1961; Goldman 2004; Sheldon et al. 1997). Note, however, that self-concept differentiation did not share unique variance with global sense of identity when it was entered simultaneously with other self-structure variables (see Table 8, Study 1b). Overlap emerged as a negative predictor for assimilation, thus indicating that greater unity in the self-concept structure was associated with less reliance on identity assimilation. It would seem that the identity processes of assimilation in itself does not serve any purpose, unless there is a dissonance within the self-concept, motivating an individual to reduce existing discrepancies. This is consistent with Whitbourne et al. (2002) descriptions of identity processes as different approaches to processing identity-discrepant experiences, different modes in dealing with changes in one’s life.

It is worth noting, that the obtained results could also be helpful for clarifying the relation between the two concepts – self and identity. From the literature review (e.g., Baumeister and Muraven 1996; Swann and Bosson 2010), one can note that the distinction between self and identity is not consistently well-established and the two concepts are sometimes used interchangeably. Since, in the present studies, the associations between self-structural indices and measures of personal identity had little predictive ability, it is tempting to assume that the way we describe ourselves in different situations or contexts, and the way we experience our selves are different phenomena. This conclusion is in line with authors who argue that the distinction between self and identity should be made and maintained (e.g., Berzonsky 2005; Katzko 2003; Oleś 2008). It should also be noticed that corresponding results, essentially no correlation between self-complexity and identity, were obtained by Suchańska and Ligocka (2011), whose study used different measures of identity, namely identity status and identity style approaches.

The results concerning the relationships of various measures of self-complexity and self-concept differentiation with thinking dispositions indicated self-concept differentiation to be the most important variable in predicting adaptive cognitive endeavors. It showed unique associations with need for cognition, reflection, and integrative self-knowledge. The direction of these effects was by no means constant and suggested that a strong, curiosity-driven, desire to engage in effortful thinking was associated with greater unity in the self-concept. In line with the above results, integrative self-knowledge appeared also to be positively related to overlap. Once again, this result indicated that an adaptive capacity to understand and integrate self-experience across time was associated with a more unified self-structure. Our results can also be interpreted as providing evidence that active cognitive processing serves as a means of uniting self-experience and reducing discrepancies within the self. As such, they support theoretical expectations derived from the existing literature (e.g., Berzonsky 2008; Campbell et al. 1996; Ghorbani et al. 2008; Njus and Johnson 2008; Trapnell and Campbell 1999).

We will sum up with two concluding remarks: (1) measures of self-complexity and self-concept differentiation do not necessary measure what they have been purported to measure, (2) results concerning the relationships of self-complexity and self-concept differentiation with adaptive outcomes are generally affected by self-contents related variables; when the confounding factors were taken into account, the true effects of structural features of the self-concept, while suggesting that psychological well-being is associated with a stable and coherent self-concept, were of minor significance.

Though the present paper is unique in its consideration of various measures of self-structure and exploring areas that had not previously been investigated (e.g., examining the impact of self-concept structure on cognitive processing), our findings are not the first to indicate that the commonly used indices of self-complexity and self-concept differentiation may lack validity (e.g., Baird et al. 2006; Locke 2003, 2006; Luo et al. 2009; Rafaeli-Mor et al. 1999; Solomon and Haaga 2003). Indeed, the reasonable degree of consensus reflected in the studies cited above argues for the invalidity, rather than the validity, of the measures employed here. Yet, these measures have been and are still being used as indices of the constructs they supposedly tap, thereby introducing potential artifacts. As it stands, there seems to be a gap between the available research evidence and using this evidence to change the measurement practice. Until this gap is filled, it would be premature to draw any definite conclusions about the relationship between the structure of the self-concept and important outcomes.

## Limitations

The current investigation has a few limitations that merit discussion. First, the present studies included Polish participants only. On the one hand, the relative homogeneity of the samples studied here was a strength, as there were likely to be fewer confounding variables. On the other hand, it raises questions regarding the generalizability of the findings, for example, whether the results would hold for individuals with different cultural backgrounds. A number of cultural psychologists have pointed out that people in collectivistic cultures (or those with a predominantly interdependent self-construal) are expected to show less cross-situational consistency in their behavior (e.g., Choi and Choi 2002; Church et al. 2008; English and Chen 2007; Markus and Kitayama 1991). Moreover, self-consistency is believed to be central to optimal functioning in individualistic cultures, but not in collectivistic cultures (e.g., Cross et al. 2003; Pilarska 2014a; Suh 2002). It should be noted that, with regard to cultural dimensions, Poland is one of the countries which Hofstede (1984) identified as an exception – while it is considered as an individualistic society, it also has high scores on both power distance and uncertainty avoidance (see also Minkov 2013; Murdoch 2009). As argued by Reykowski (1994, 1998), despite popular belief that there has been a major change toward individualism in Poland, strong collectivistic elements have persisted.

There was also another limitation in relation to the samples. Given the developmental changes taking place during emerging adulthood (i.e., ages 18–25; Arnett 2000), the inclusion criteria could have been stricter or age could have been introduced as an additional independent variable. Since identity exploration – an active experimentation with different social roles – is thought to be an important feature of emerging adulthood (e.g., Arnett 2000; Schwartz et al. 2005), there may be a theoretical reason to expect age-related differences in the structure of self-concept among our participants. Diehl et al. (2001) obtained some evidence that self-concept differentiation was related to age, and that the association between self-concept differentiation and psychological well-being was moderated by age. More precisely, the negative effect of self-concept differentiation on psychological well-being was more pronounced in older adults than in younger adults.

Finally, an additional limitation of our studies could be the use of the Self-Incoherence Scale. This measure is similar to the one used by Donahue et al. (1993), but with considerably fewer adjectives, all of which are positively valenced. Since the indices of self-concept differentiation are sensitive to the within-role variation and the mean trait rating, perhaps using a longer and more balanced list of traits could reduce (but by no means eliminate) their limitations.

## Recommendations

The conflation of self-concept structure and self-concept content evidenced by our results not only presents a problem for research, but also challenges the underlying theoretical models themselves. Both Linville’s (1987) and Donahue et al.’s (1993) models assume that structure and content are independent and, more specifically, that the valence of self-content is unrelated to structure. While various authors have reported on the inability to distinguish between structure and content (valence) by the applied measures, the possibility that the assumptions behind these models may themselves be the problem has been given less attention. Yet, research by Woolfolk and colleagues (1995, 2004) demonstrated that evaluative valence may affect self-complexity, and identified two partially independent dimensions of self-complexity, namely positive self-complexity and negative self-complexity. In a similar vein, Locke (2006) provided support for the relative independence of positive and negative self-concept differentiation. The possible effects of features of the self-knowledge, other than valence, on the structure of the self-concept seem to deserve further investigation. Meanwhile, whether it is just that the currently available self-structural measures are vulnerable to self-enhancement and social desirability biases (e.g., the over-endorsement of positive traits) or the contents of the self-concept have an influence on the way they are organized, the inclusion of a measure of self-esteem should be considered standard practice in future studies utilizing measures of self-structure.

Moreover, the search for potential moderators of the effects of self-concept structure should continue. Only a few previous studies (besides those mentioned earlier) have examined whether the relationship between the structure of the self-concept and psychological well-being was conditional on other variables. McConnell et al. (2005) found support for a moderating role of self-aspects control, meaning that the positive relation between self-complexity and poor well-being was evident among those with low perceived control over their self-aspects. In another study, McConnell et al. (2006) found interactions between self-complexity and three of the Big Five major personality traits (openness, conscientiousness, and agreeableness) in accounting for differences in well-being. The findings of Diehl and Hay (2011) revealed that the relationship of self-concept differentiation and well-being was qualified by self-concept clarity. Other relevant factors that might moderate the effects of self-concept structure could include, for example, importance (centrality) of one’s self-aspects and internalization of one’s self-aspects (Ryan and Deci 2003).

Future research should also examine potential antecedents of different dimensions of self-structure. There is very little empirical evidence on what factors actually lead to individual differences in the self-concept structure. To our knowledge, except for cross-cultural studies, there have been only few investigations on this topic. Using a developmental perspective, Evans and Seaman (2000) proposed that individual differences in self-complexity could be explained by differences in maturity of defense mechanisms. Also encouraging are the findings by Lutz and Ross (2003) that link self-concept differentiation to aspects of parental bonding. Moreover, conclusions drawn from the longitudinal study of Donahue et al. (1993) support the view that psychological adjustment may be a causal antecedent to self-concept differentiation.

## Footnotes

1 We used Styła et al.’s (2010) tool called Self-Incoherence Scale to assess self-concept differentiation. Therefore, the terms self-concept differentiation and self-incoherence will be used interchangeable throughout this paper.

2 In computing the SCD_SD_ score, we allowed for up to seven non-responses (20 %), with no more than one omission on each trait adjective. When calculating SCD indices based on correlation coefficients, we allowed for one role (20 %) to be excluded from the correlation matrix, either because of the zero within variance or because of non-responses on all seven trait adjectives. The remaining six correlations were then averaged or processed by factor analysis to obtain the SCD_R_ and the SCD_VAR_ scores, respectively.

3 We allowed for up to 20 % of non-responses in each subscale and then used a single imputation procedure (person mean substitution). Missing values were replaced with the intraindividual mean of the other items on that subscale. Missing responses to the other multi-item measures used in this study were treated in the same manner unless otherwise specified.

4 A total of 17.3 % of the sample received a zero score on OL thereby causing a divide by zero in further computation of the SC statistic.

5 We performed additional regression analyses to examine the potential confounding of quantity with evaluation. Both the number of chosen adjectives and the proportion of positive traits chosen (PPAT) were entered to assess whether they uniquely contributed to the prediction of the dependent variables. The results showed that the number of attributes uniquely contributed to the prediction of NSA (β = 0.47, *p* < 0.001), OL (β = 0.25, *p* < 0.001), and the H score (β = 0.97, *p* < 0.001), and the proportion of positive attributes uniquely predicted OL (β = 0.11, *p* < 0.01) and the H score (β = 0.03, *p* < 0.05). Sakaki’s SC statistic was unrelated to any of these two variables (β = 0.03 and β = −0.04, *ns*, respectively). It is worth noting that the richness of self-depiction and its favorability were not exactly independent of each other, as demonstrated by a significant correlation between them (*r* = −0.18, *p* < 0.001). Moreover, in the model of the H score, the interaction term of NAT and PPAT appeared to be significant (*F*(1, 648) =8.63, *p* < 0.01). The effect of the number of traits on the H score was consistently positive and increased with increasing proportion of positive traits.

6 We also employed Hayes (2012) bootstrapping PROCESS tool for SPSS to assess the direct and indirect effects of NSA and OL on either the H or SC scores, with NAT as a mediating variable. The remaining component of self-complexity was used as a covariate and partialed out of all paths in the respective model. A total of 1000 re-samples of the data were executed using Hayes’ SPSS macro. The confidence intervals for the indirect effects of NSA and OL on the H scores via NAT did not include zero (estimate =0.12, 95 % CI =0.09, 0.14 and estimate =0.73, 95 % CI =0.40, 1.02 for NSA and OL, respectively). Thus, the number of traits mediated the relationships of the number of self-aspects and overlap with the H score. The indirect effect of NSA on the SC score was found to be significant (estimate = −2.89, 95 % CI = −6.70, −0.87). However, there was no indication of a significant indirect effect of overlap on the SC scores through the number of traits (estimate = −4.07, 95 % CI = −18.78, 1.56).

7 For a total of 7.2 % of the sample, the SCD_R_ and SCD_VAR_ scores could not be calculated because of zero within variation for more than one role.

8 The comparison of all three groups (low, medium, and high mean trait ratings) led to the same conclusions. According to Kruskal-Wallis tests, there was a significant effect of the mean self-rating classification on the SCD_SD_ score (χ2(2) =129.62, *p* < 0.001, E_*R*_^2^ = 0.15). Subsequent pairwise comparisons, performed using the Mann–Whitney U test, showed that all three groups were significantly different from each other with respect to the SCD_SD_ scores (*p* < 0.01).

9 Bootstrapping analysis (Hayes 2012) was used to test the mediating role of the mean trait rating. A total of 1000 re-samples of the data were executed using Hayes’ macro. As zero was not in the 95 % confidence interval for the indirect effect, we can conclude that it was indeed significant (estimate = −0.12, 95 % CI = −0.24, −0.03).

10 In each of the samples, we made a series of comparisons between participants for whom we had data and those for whom we did not, to assess whether there was any notable difference between them on any of the outcome measure. Comparisons were performed using the Mann–Whitney U-test. Significant differences were found between those for whom we obtained the SCD_VAR_ and SCD_R_ scores and those for whom we did not in relation to global sense of identity (Study 1a: *U* =1784.00, *p* < 0.01, *r* = 0.15); and between those for whom we obtained the SC scores and those for whom we did not in relation to accommodation (Study 1c: *U* =436.50, *p* < 0.05, *r* = 0.19) and balance (Study 1c: *U* =299.50, *p* < 0.001, *r* = 0.30).

11 The PROCESS macro (Hayes 2012) was used to test for mediation and moderation effects. We found evidence for mediation of the relationship between self-concept differentiation and global sense of identity, through the mean trait ratings (estimate = −0.11, 95 % CI = −0.17, −0.06 and estimate = −0.10, 95 % CI = −0.14, −0.06 for the indirect effects of SCD_SD_ in Study 1a and Study 1b, respectively).

12 There were significant differences with regard to the cognitive variables among participants for whom we had complete data and those for whom we did not, suggesting that a bias would be present. Those for whom we did not obtain the SCD_VAR_ and SCD_R_ scores had lower levels of integrative self-knowledge than did those for whom we could calculate both scores (*U* =9722.50, *p* < 0.05, *r* = 0.11). The same was true in case of the SC scores (*U* =16487.00, *p* < 0.01, *r* = 0.13).

13 Using PROCESS bootstrapping macro (Hayes 2012), we found that the effect of self-concept differentiation on need for cognition as well as on integrative self-knowledge was mediated by the mean trait ratings (estimate = −3.89, 95 % CI = −5.79, −2.42 and estimate = −0.09, 95 % CI = −0.15, −0.04 for the indirect effect of SCD_SD_ on NCS and ISK, respectively). Also, the number of traits used in the sort served as a significant mediator between overlap and integrative self-knowledge (estimate =0.13, 95 % CI =0.03, 0.28 for the indirect effect of OL).

14 In a private correspondence, Michiko Sakaki herself has suggested the use of overlap and the number of aspects participants reported as alternative measures of self-complexity. One strategy, that we felt might be useful for overcoming division by zero would be adding a small constant (for example, 0.001) to the divisor. The majority of previous findings were replicated when this correction was used, except that the correlation between accommodation and the SC score was lost (Study 1c, *r* = 0.18, *ns*), whereas the correlation between balance and the SC score was significant (Study 1c, *r* = −0.27, *p* < 0.01). However, in the simultaneous regression, the SC score was unrelated to either accommodation or balance (β = 0.14 and β = −0.10, *ns*, respectively). Full results are available on request.
